# Prevalence of severe acute respiratory syndrome coronavirus 2 spike antibodies in some healthcare settings in Egypt

**DOI:** 10.1186/s42506-022-00106-4

**Published:** 2022-06-04

**Authors:** Engy Mohamed El-Ghitany, Azza Galal Farghaly, Shehata Farag, Mona H. Hashish, Fahmy Charl, Eman A. Omran

**Affiliations:** 1 Department of Tropical Health, High Institute of Public Health, Alexandria University, 165 El-Horreya Avenue, Alexandria, Egypt; 2 Department of Biostatistics, High Institute of Public Health, Alexandria University, 165 El-Horreya Avenue, Alexandria, Egypt; 3 Family and Community Medicine Department, Faculty of Medicine, King Khalid University, Abha, Saudi Arabia; 4 Department of Microbiology, High Institute of Public Health, Alexandria University, 165 El-Horreya Avenue, Alexandria, Egypt; 5 Department of Occupational Health and Industrial Medicine, High Institute of Public Health, Alexandria University, 165 El-Horreya Avenue, Alexandria, Egypt

**Keywords:** Anti-spike, Healthcare workers, COVID-19, Serosurveillance

## Abstract

**Background:**

Healthcare workers (HCWs) are at the front line in battling infection transmission, such as that in coronavirus disease 19 (COVID-19). Additionally, they may act as potential carriers passing the virus on to others. Anti-spike (anti-S) antibodies for severe acute respiratory syndrome coronavirus 2 (SARS-CoV-2) are formed either as a result of infection or vaccination with both indicating immunity against future COVID-19 infection.

**Aim:**

This study aimed to identify the prevalence of COVID-19 seropositivity among HCWs.

**Methods:**

This cross-sectional study included 559 HCWs from 39 hospitals with variable degrees of COVID-19 exposure risk (depending on the occupation, department, and hospital type). Demographic data were recorded as well as history of COVID-19 infection and vaccination. Serum samples were collected and tested for SARS-CoV-2 spike antibodies.

**Results:**

Anti-S positivity was found in 59.0% of the participating 559 HCWs, indicating a high level of seroprotection. Of the 559 HCWs, 34.1% had reported previous infection with COVID-19. Following infection, only 46 (24.0%) of those affected received vaccination. Anti-S seropositivity was found in 39.1% of participants who were unvaccinated and had no history of infection. Physicians had the highest median anti-S titers (58.0 relative units (RU)/mL), whereas pharmacists and office staff had the lowest (25.7 and 38.2 RU/mL, respectively).

**Conclusions:**

Overall, 59.0% of the 559 HCWs were anti-S positive, indicating a relatively high seroprotective status. Among those who were unvaccinated and had no history of infection, 39.1% were seropositive for anti-S, denoting a high rate of silent/asymptomatic infections. Screening of HCWs for SARS-CoV-2 anti-S is recommended, along with the vaccination of seronegative individuals.

**Supplementary Information:**

The online version contains supplementary material available at 10.1186/s42506-022-00106-4.

## Introduction

Coronavirus disease 19 (COVID-19) is a global pandemic wreaking havoc on public health. This pandemic has hit Egypt, with the first confirmed case officially announced on February 14, 2020 [1]. The related morbidity and mortality of severe acute respiratory syndrome coronavirus 2 (SARS-CoV-2) necessitate the estimation of its prevalence among various high-risk groups [2]. The potential spread of illness among frontline healthcare workers (HCWs), who themselves are at risk while also acting as a reservoir of infection to their patients, colleague HCWs, and domestic family members, is a worldwide concern [3]. Asymptomatic infections in this vulnerable risk group pose a higher magnitude of the problem and higher transmission and mortality rates [4]. In one study, the risk of contracting COVID-19 was three times higher for patient-facing HCWs than for non-patient-facing HCWs and the general population. Additionally, HCWs had a sevenfold higher chance of contracting severe COVID-19 than non-HCWs [5].

HCWs are exposed to long working hours under significant pressure and insufficient resources (e.g., lack of personal protective equipment [PPE] and infrequent screening for COVID-19 exposure or infection clinical testing), in addition to the risk of close contact with patients with high viral load, due to the pandemic situation as an emergency. Furthermore, additional factors such as sleep deprivation, stress, and circadian inconsistency may impact their immune system, making them more susceptible to infection [3, 5].

Serosurveillance is a strategy of epidemiology that seeks to monitor the dynamics of disease transmission using serological tests (antibody testing) to generate data on the prevalence of infection and immunity in population groups. This can help to determine the level of antibodies required to achieve herd immunity and identify groups of susceptible individuals. It is also used to evaluate the duration of protective antibodies and determine the effectiveness of vaccination [6].

In the healthcare settings, serosurveillance studies are specifically needed to discover asymptomatic infections among HCWs, evaluate the utilization of PPE and infection control measures, identify risk factors for infection, assist policymakers in reducing infections in the healthcare settings, and assess vaccination coverage. The factors affecting COVID-19 exposure and seropositivity of HCWs include their occupation, the hospital type (whether dealing with patients with COVID-19 or not), and department in which HCWs are located [6, 7]. Therefore, prevention and control measures could be more targeted in health facilities based on the availability of institution-based risk stratification.

Several commercial SARS-CoV-2 antibody tests use specific viral antigens, with the spike protein being the most important. Anti-spike (anti-S) positivity denotes either previous infection or vaccination and indicates relative immunity against future reinfection [8].

This study was performed to assess the magnitude of SARS-CoV-2 anti-S among a group of HCWs (including vaccinated and previously infected ones), which would reflect their immune status against future reinfections. This study also elucidated the relation between the occupation of HCWs, hospital type, and department and the SARS-CoV-2 anti-S seropositivity.

## Methods

### Study design

This cross-sectional survey was conducted throughout the period between January and June 2021. This period coincided with the second and third waves of the COVID-19 pandemic in Egypt.

### Sample size calculation

A total sample size of 448 HCWs was required to estimate the mean prevalence of COVID-19 antibodies of 70%, with precision confirmation of 6% at 95% confidence level and a design effect of two. Following an extensive literature review, the sample size was estimated using Epi Info 7 software with the cited values [9]. A total of 559 participants were included in the study to increase the confidence of our estimate and have a greater precision.

### Selection criteria

The HCWs included physicians, nurses, technicians, pharmacists, employees, office personnel, personal care workers, and faculty members. Our inclusion of such participants was based on the classification of the World Health Organization (WHO) for HCWs [10]. HCWs who declined to participate (via either blood sampling or questionnaire interview) were excluded from the study. Participants who had been vaccinated and those who had previously been infected with COVID-19 were included in the study. Previous history of COVID-19 infection was based on one or more of the following criteria: clinical symptoms, radiological results, and laboratory test results). The Egyptian national guidelines were used to determine the criteria for diagnosing infected cases.

### Sampling technique

A total of 39 hospitals were randomly selected from 5 governorates using the multistage cluster sampling technique. At the first stage, the provinces and governorates were also chosen randomly using STATA software by adding the province and governorate names. The included provinces were Greater Cairo, Alexandria, and Delta. At the second stage, 14 government hospitals, 10 university hospitals, 8 private hospitals, and 7 medical insurance hospitals from the selected governorates were included. The number of each type of these hospitals was estimated to be proportionally allocated to the number of different types of hospitals in Egypt. A high number of hospitals (*n* = 39) were included to fulfill our sample size because several HCWs either declined or were attending to their patients and thus could not participate. A convenient sampling of HCWs was performed until the sample size was fulfilled.

### Data collection methods and tools

A structured interview questionnaire sheet was designed and filled in for each participant. It included data on the occupation, hospital type, department name, history of COVID-19 diagnosis, and vaccination.

A pilot study was conducted before research implementation to test for the feasibility of recruitment as well as validation of the questionnaire. This was performed on a group of 20 HCWs in one of the university hospitals.

A 3-mL venous blood sample was collected from each participant for antibody testing. Serum samples were then separated by centrifugation at 3000 rpm and stored at − 20 °C until further processing. All samples were tested for anti-S.

The anti-SARS-CoV-2 QuantiVac sandwich enzyme-linked immunosorbent assay (ELISA) technique (EUROIMMUN, Lübeck, Germany) was used to detect the immunoglobulin class IgG against the S1 domain of the viral spike protein. According to the manufacturer's instructions, the results should be interpreted according to their relative unit (RU) results as follows: the titers of < 8 RU/mL were considered negative, those of ≥ 8 to < 11 RU/mL were considered borderline, and those of ≥ 11 RU/mL were considered positive. However, for the purpose of this study, the borderline results were considered positive for more accessible statistical analysis. Quantitative results were also expressed as quartiles because some samples had readings exceeding the highest calibrator in the kit (> 120 RU/mL), and thus, quartiles were used for correlations with quantitative variables.

### Statistical analysis

After data extraction, these were revised, coded, and fed to IBM SPSS version 22 statistical software (SPSS Inc., Chicago, IL). All statistical analyses were performed using two-tailed tests. A *p* value of < 0.05 was considered statistically significant. The frequency and percent distribution of descriptive analysis was performed for all variables, including sociodemographic data, screening results, and immunity status. On the basis of the sensitivity and specificity of the used kits, as stated by their manufacturers, the adjusted prevalence rates were calculated using the crude prevalence rates to adjust any false-positive or false-negative results [11]. Cross-tabulation was performed to test some relations with serological findings among HCWs. The significance of relations was determined using an exact probability test for small frequency distribution.

## Results

A total of 559 HCWs were included. The majority of participants (85.2%) were from Alexandria Governorate and urban areas (95.2%), and 73.5% were women. The most common age group was 30–39 years (36.1%), followed by 50+ years (29.2%). The majority of HCWs (62.3%) were from general, multipurpose hospitals, whereas 18.4% were from COVID-19 isolation hospitals and 14.8% were from hospitals performing screening for COVID-19. Only 11.1% of participants were from hospitals not dealing with patients with COVID-19. Regarding occupation, the majority of HCWs were physicians (42.8%), whereas 17.9% were nurses and 12.2% were pharmacists (Supplementary Table 1).

HCWs were categorized into four groups as follows: neither vaccinated nor previously infected (*n* = 271 [48.5%]), previously infected and unvaccinated (*n* = 145 [25.9%]), vaccinated and not previously infected (*n* = 97 [17.4%]), and vaccinated with a history of infection prior to vaccination (*n* = 46 [8.2%]). The overall anti-S positivity among the 559 HCWs was 59.0% (83.9% among vaccinated and 50.5% among unvaccinated) (Table 1).

**Table 1:** Prevalence of anti-S positive results among 416 unvaccinated HCWs according to their occupation, Egypt, Jan-June 2021

Occupation	Crude prevalence %	Adjusted^**a**^ prevalence %	95% CI	
			LL%	UL%
Physicians	48.6	52.1	44.1	60.1
Nurses	57.8	62.0	52.0	72.0
Technicians	48.1	51.6	38.0	65.2
Pharmacists	49.0	52.5	38.8	66.2
Personal care workers	69.2	74.2	51.3	97.1
Office staff	42.6	45.6	33.1	58.1
**Overall**	59.0	63.3	59.3	67.3

^a^Adjustment of prevalence was done based on sensitivity and specificity of the test kit, as mentioned by the manufacturer

The highest crude prevalence for anti-S results according to department was for those working in radiology departments (66.7%; 95% confidence interval (CI), 48.7–94.3%), followed by wards (58.6%; 95% CI, 50.4–75.2%). Conversely, the lowest crude prevalence for anti-S results according to department was recorded among HCWs working in infection control units (22.2%; 95% CI, 1.3–51.5%) and blood banks (25%; 95% CI, 1.5–70.1%) (Table 2).

**Table 2:** Prevalence of anti-S positive results among 416 unvaccinated HCWs according to their work department inside hospitals Egypt, Jan-June 2021

Work department	Crude prevalence %	Adjusted^**a**^ prevalence %	95% CI	
			LL%	UL%
Radiology	66.7	71.5	48.7	94.3
Wards	58.6	62.8	50.4	75.2
Intensive care units	57.1	61.2	45.1	77.3
Intervention room	56.3	60.4	43.5	77.3
Emergency department	54.3	58.2	41.9	74.5
Surgical theaters	51.7	55.4	37.3	73.5
Pharmacies	51.2	54.9	39.7	70.0
Laboratory	45.6	48.9	37.9	59.9
Administrative offices	44.4	47.6	36.1	59.1
Outpatient clinics	44.3	47.5	36.5	58.5
Blood banks	25.0	26.7	1.5	70.1
Infection control units	22.2	23.7	1.3	51.5

^a^Adjustment of prevalence was done based on sensitivity and specificity of the test kit, as mentioned by the manufacturer

The highest crude prevalence for anti-S results according to the hospital type was among those working in pulmonology hospitals (65.7%; 95% CI, 55.4–85.6%), followed by HCWs working in hospitals performing screening for COVID-19 (56.2%; 95% CI, 49.1–71.5%). Conversely, seropositivity for anti-S was lowest among HCWs working in outpatient clinics (37.5%; 95% CI, 6.2–74.2%) (Table 3).

**Table 3:** Prevalence of anti-S positive results among 416 unvaccinated HCWs according to the type of hospital, Egypt, Jan-June 2021

Type of hospital	Crude prevalence%	Adjusted^**a**^ prevalence%	95%CI	
			LL%	UL%
Pulmonology hospital	65.7	70.5	55.4	85.6
Performing screening for COVID-19	56.2	60.3	49.1	71.5
Not dealing with COVID-19	55.8	59.8	46.5	73.1
General (receives COVID-19 and other patients)	52.3	56.1	49.9	62.3
COVID-19 isolation	41.8	44.8	33.8	55.8
Outpatient clinics	37.5	40.2	6.2	74.2

^a^Adjustment of prevalence was done based on sensitivity and specificity of the test kit, as mentioned by the manufacturer

Among HCWs who neither were vaccinated nor had a previous infection, 60.9% were negative for anti-S, whereas 39.1% were positive for anti-S. Among HCWs who had a history of COVID-19 infection and no vaccination, 28.3% were negative, whereas 71.7% were positive. Vaccinated HCWs who reported no previous history of infection showed 77.3% anti-S positivity, and this value increased to 97.8% in the group who reported having both vaccination and previous infection. Significant differences were recorded among the anti-S results in the four groups (*p* = 0.001) (Table 4).

**Table 4:** Anti-S IgGs among HCWs according to history of COVID-19 infection and vaccination, Egypt, Jan- June 2021

SARS-CoV-2 antibody test	Unvaccinated (***n*** = 416)				Vaccinated (***n*** = 143)				
	No history of COVID-19 infection		With history of COVID-19 infection		No history of COVID-19 infection		With history of COVID-19 infection		
	No.	%	No.	%	No.	%	No.	%	
**SARS-CoV-2 anti-S (*n*** **= 559)**									0.001*
Negative	165	60.9	41	28.3	22	22.7	1	2.2	
Positive	106	39.1	104	71.7	75	77.3	45	97.8	

*P* exact probability test

*p < 0.05 (significant)

**Fig. 1:**
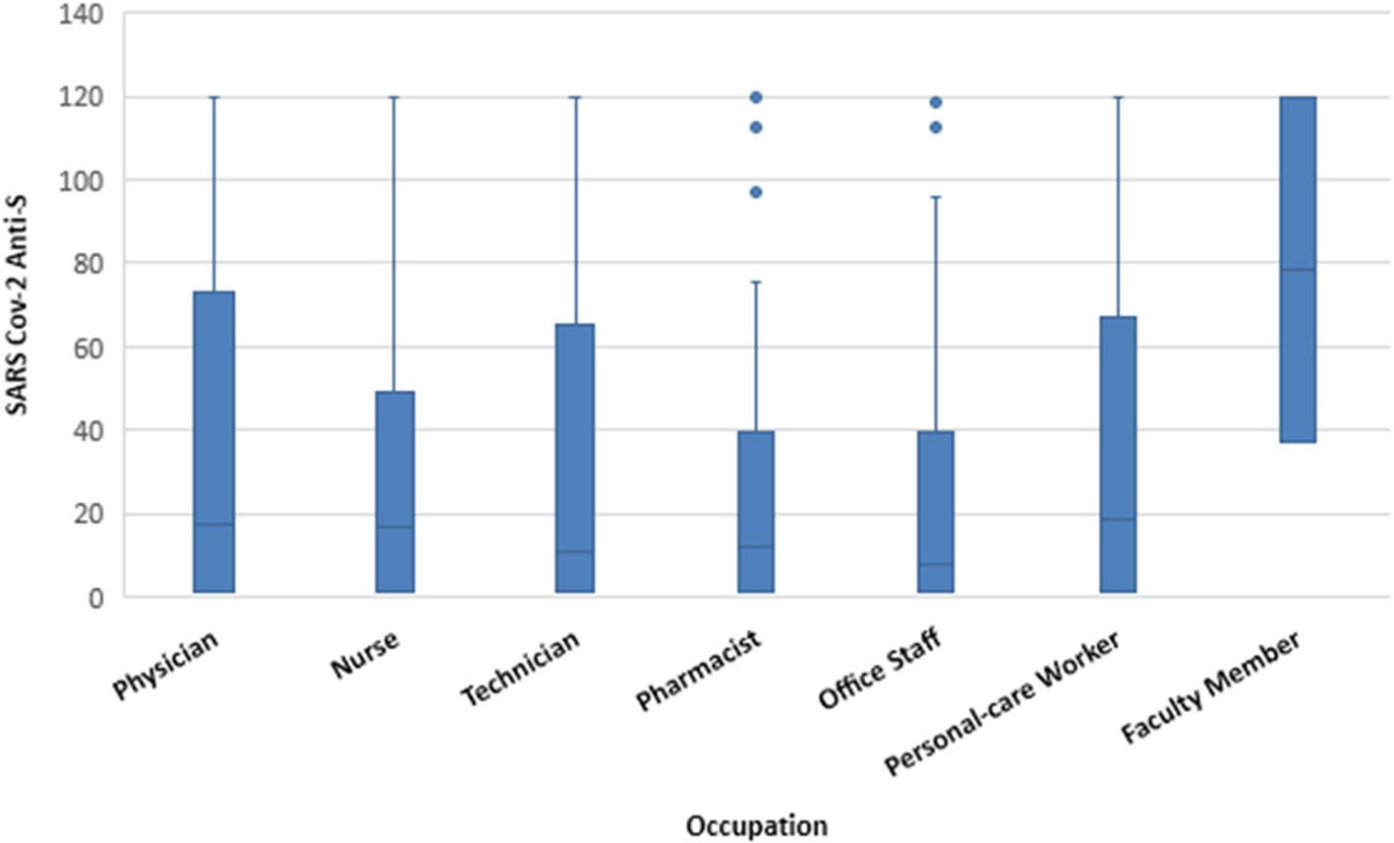
Box-Plot for SARS-CoV-2 anti-S titers (RU/ml) quartiles among anti-S positive HCWs by type of occupation, Egypt, Jan - June 2021

**Fig. 2:**
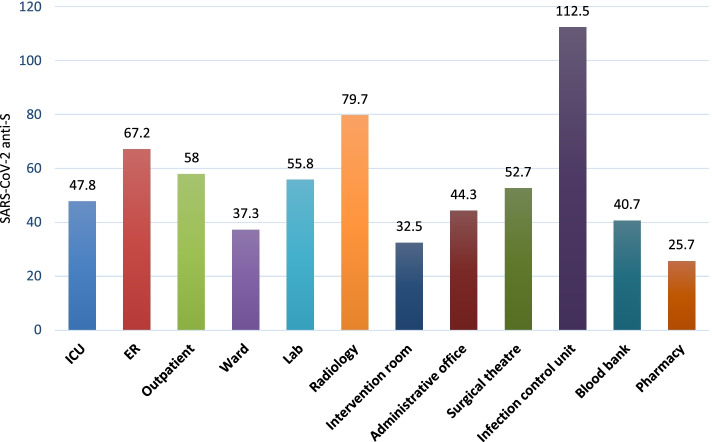
Median SARS-CoV-2 anti-S titers among anti-S positive HCWs by hospital department, Egypt, Jan-June 2021

Regarding occupation, physicians had the highest median anti-S titers (58.0 RU/mL). The lowest titers were found among pharmacists and office staff (25.7 and 38.2 RU/mL, respectively) (*p* = 0.254) (Fig. 1). Regarding hospital department, the highest median anti-S titers were found among those working in infection control, radiology, and emergency departments (112.5, 79.7, and 67.2 RU/mL, respectively). In contrast, the lowest median titers were found among those working in hospital pharmacies (Fig. 2 and Supplementary Table 2).

## Discussion

Although people of all ages and occupations can be infected with SARS-CoV-2, HCWs probably have the highest risk of contracting the infection [4, 7, 9]. The asymptomatic and presymptomatic portions of SARS-CoV-2 infections are estimated to be up to 30% [12]. The anti-spike status of HCWs can reflect acquired immunity resulting either from exposure or vaccination. The seroconversion rate among frontline providers could provide valuable information regarding the effectiveness of the PPE strategy [13]. The current study was conducted to assess SARS-CoV-2 anti-spike IgG seroprevalence among a group of HCWs. Differences in seroprevalence of anti-spike frequency regarding hospital type, department, and occupation were also investigated.

Of the 559 HCWs, 34.1% had a positive history of COVID-19 infection. Although this percentage is higher than several other reported results (3.7 to 20%) in Egypt [4, 14, 15], it is consistent with that recorded by El-Sokkary et al. (yet their sample size was smaller [*n* = 82 HCWs], all were working in isolation hospitals) [16]. Two studies, one in Germany [17] and the other in the Netherlands [18], reported much lower infection rates among HCWs (5.4% and 1%, respectively), reflecting strict adherence to infection control measures. High infection rates among HCWs, if proven to be work-related, are attributed to their higher exposure as well as a need for more diligent infection control measures and the use of PPE. The WHO recommends guidelines for PPE use in the COVID-19 pandemic, including universal masking, with respirator (N95 or equivalent) utilization only during approved aerosol-generating procedures [19]. However, the implementation of such guidelines is often limited by resource availability. Comparisons of the COVID-19 infection rates among HCWs across countries may not be valid because of the differences in health system structure and organization, availability and enforcement of healthcare protocols and PPE, and differences in declared COVID-19 prevalence rates between countries [20].

Only 18.4% of HCWs included in our study were from COVID-19 isolation hospitals. This relatively small proportion may explain the low vaccination rate (25.6%) among our HCWs since at the time of our study; vaccination was prioritized, when available, for HCWs in isolation hospitals. Currently, several vaccines are readily available for all HCWs and the general population.

Vaccinated HCWs who reported no previous history of infection showed 77.3% anti-S positivity, and this value increased to 97.8% in the group who reported having both vaccination and previous infection. This highlights the hybrid immunity offered by vaccination and natural infection compared with that by vaccination alone [21].

Only 24.0% of those with previous COVID-19 infection received vaccination following infection, whereas the rest did not. The reasons for not obtaining the vaccine following infection may be the lack/delay of vaccine delivery at the time of the study (local vaccine manufacturing had not yet started at that time) or lack of confidence of the HCWs in the importance of vaccination following infection. Guidelines in vaccine delivery recommend that, following COVID-19 infection, a period of 90 days passes before vaccination if monoclonal antibodies or convalescent plasma were received; otherwise, 14 days is sufficient. Those factors may have contributed to the low vaccination rate among those with previous infections. The reasons for not taking the vaccine were not tackled in the current study.

The overall anti-S positivity among the 559 HCWs was 59.0%, denoting a relatively high seroprotective status. This high rate might be over-estimated due to the inclusion of borderline along with the positive anti-S cases. However, this inclusion was important owing to the importance of borderline results and their partial protective role against COVID-19 infection. Such inclusion might explain our high seroprevalence rates compared to other similar studies.

Among those who neither were vaccinated nor had a history of infection, 39.1% were seropositive for anti-S, denoting the high rate of silent and asymptomatic infections. Slightly higher rates (40.3%) were even reported in a similar study by El-Sokkary et al. in Egypt among unvaccinated HCWs [16], but all of those were HCWs in isolation hospitals only. In contrast, our study included heterogeneous hospital types; therefore, our rate would be considered high for such hospital types. Nevertheless, lower rates were reported in a study in Saudi Arabia (14.8%) [16]. Our high rates emphasize the importance of serosurveillance for anti-S among HCWs, not merely counting on screening for clinical symptoms of COVID-19. Serosurveillance is necessary for several reasons, including the prevention of complications among vulnerable individuals and assessment of infection control measures in healthcare facilities.

Several studies have investigated the work related factors in hospitals for anti-S seropositivity. Piccoli et al. reported that seropositivity was significantly higher in high-risk occupations (i.e., physicians and nurses) than in low-risk ones (i.e., personal care workers and administrative staff) (10.11% vs. 6.56%; odds ratio (OR) = 1.75) [20]. A study in Egypt reported that SARS-CoV-2 seropositivity was associated with being a physician and exposure to patients with COVID-19 in isolation hospitals for longer durations (> 3 months) [16]. In our study, the crude prevalence for anti-S seropositivity among unvaccinated physicians was not high compared with that in other occupations (48.6%; 95% CI, 44.1–60.1%); however, they had the highest median anti-S titers (58.0 RU/mL). Together, these findings suggest that personal care workers, nurses, and pharmacists have higher probabilities to contract the infection, but physicians are more likely to have higher viral loads. Our findings did not reach statistical significance (*p* = 0.254) but may be proven in other studies with larger sample sizes. This indicates that physicians may be exposed to higher viral loads because of their direct and repeated contact with patients with COVID-19, with higher probabilities of severe infection due to high viral load. High antibody levels indicate exposure to high viral loads. Hospital office staff and pharmacists do not carry the same risk as physicians of high viral load do because of their less risky exposure to patients. Physicians should use more PPE and infection control measures.

Regarding the department in the hospital, differences in titers among departments did not reach statistical significance (*p* = 0.069). Radiology departments could be considered high-risk areas in hospitals because of the repeated exposure to patients with COVID-19 during the performance of chest imaging. Emergency departments may also pose a risk for HCWs contracting COVID-19 because of this department’s “emergency” nature, where effective infection control measures taken by patients may not be promptly adequate. These data help to stratify the risks of COVID-19 exposure inside hospitals according to the nature of departments. More diligent measures should be taken in departments with high seroprevalence. Piccoli et al. reported that the association between hospital site and SARS-CoV-2 seroprevalence was no longer significant (*p* = 0.932) after mutual adjustment for ward/unit type and HCW occupation [20].

Our highest crude prevalence for anti-S results was among those working in pulmonology hospitals (65.7%; 95% CI, 55.4–85.6%), whereas the lowest was among HCWs working in outpatient clinics (37.5%; 95% CI, 6.2–74.2%). Similarly, Scozzari et al. [21] documented that HCWs in COVID-19 wards showed higher anti-S frequency than those in non-COVID-19 wards. A study reported that HCWs with the most exposure to patients with COVID-19 were not at higher risk for developing anti-S than those with limited COVID-19 exposure, and the authors attributed this to their strict institutional adherence to the WHO guidelines for PPE use [13]. Despite efforts to protect high-risk first-line HCWs, more infection control measures should still be taken.

### Study strengths and limitations

To the best of our knowledge, similar studies on seroprevalence of SARS-CoV-2 among HCWs are limited in Egypt. This study also investigated the anti-S titers in relation to the hospital type, occupation, and department. Higher anti-S titers indicate exposure to higher viral loads (among unvaccinated individuals), highlighting the inadequacy in the use of PPE.

The COVID-19 vaccination first became available only during participant recruitment and not before research initiation. Because the situation has changed considerably in recent months, the vaccination coverage indicated in this article no longer reflects the current picture. Borderline cases were included with the positive ones in the statistical analysis, and this may explain the higher prevalence rate compared with those reported by other studies. Governorates included in our study were from Lower Egypt, limiting the generalization of our results. Further studies including HCWs from Upper Egypt governorates would provide a more comprehensive view of the overall seroprevalence in Egypt.

## Conclusions

HCWs in our study had an overall 59.0% anti-S seroprevalence rate including those who received COVID-19 vaccines. Anti-S seroprevalence reached 83.9% among vaccinated HCWs (some received only one dose at recruitment time), and it was 50.5% among unvaccinated HCWs. Among those who neither were vaccinated nor had a history of infection, 39.1% were seropositive for anti-S, denoting the high rate of silent and asymptomatic infections. The prevalence may even be higher after adjustment. Personal care workers, those working in pulmonology hospitals and radiology units, had the highest anti-S prevalence rates.

Screening of HCWs for SARS-CoV-2 anti-S is recommended together with vaccination of seronegative individuals. Completion of vaccine doses of all HCWs is recommended for full protection. Special attention should be paid to HCWs at higher risk of contracting COVID-19 infection, including those in high-risk departments or occupations.

## Supplementary Information

**Additional file 1: Supplementary Table 1.** Socio-demographic data of 559 health care workers, Egypt, 2020. Supplementary Table 2: Distribution by quartiles of SARS-CoV-2 anti-S titres among 416 unvaccinated SARS-CoV-2 anti-S positive HCWs

**Additional file 2.**

## Data Availability

The datasets used and/or analyzed during the current study are available from the corresponding author on reasonable request.

## Abbreviations

ELISA: Enzyme-linked immunosorbent assay
HCWs: Healthcare workers
IRB: Institutional Review Board
PPE: Personal protective equipment
RU: Relative unit
WHO: World Health Organization
