# Supplementary material for: Prevalence of severe acute respiratory syndrome coronavirus 2 spike antibodies in some healthcare settings in Egypt
Source: J Egypt Public Health Assoc. 2022 Jun 4;97:11. doi: 10.1186/s42506-022-00106-4 (PMC9166239; doi:10.1186/s42506-022-00106-4)
Supplement: Supplementary file 1 — Additional file 1: Supplementary Table 1. Socio-demographic data of 559 health care workers, Egypt, 2020. Supplementary Table 2: Distribution by quartiles of SARS-CoV-2 anti-S titres among 416 unvaccinated SARS-CoV-2 anti-S positive HCWs [file 42506_2022_106_MOESM1_ESM.docx]

**Table 1 (Supplementary): Socio-demographic data of 559 health care workers, Egypt, 2020**

| **Socio-demographic data** | | **No** | **%** |
| --- | --- | --- | --- |
|  | Giza | 59 | 10.6% |
| **Governorate** | Alexandria | 476 | 85.2% |
|  | Menoufeya | 4 | 0.7% |
|  | Cairo | 3 | 0.5% |
|  | Kafr El-Sheikh | 17 | 3.0% |
| **Sex** | Female  Male | 411  148 | 73.5%  26.5% |
| **Age ( years)** | < 30 | 63 | 11.3% |
|  | 30-39 | 202 | 36.1% |
|  | 40-49 | 131 | 23.4% |
|  | 50+ | 163 | 29.2% |
| **Residence** | Urban | 532 | 95.2% |
|  | Rural | 15 | 2.7% |
|  | Slum | 12 | 2.1% |
| **Occupation** | Physician | 239 | 42.8 |
|  | Nurse | 100 | 17.9 |
|  | Technician | 59 | 10.6 |
|  | Pharmacist | 68 | 12.2 |
|  | Worker | 14 | 2.5 |
|  | ambulance driver | 1 | 0.2 |
|  | Office Staff | 72 | 12.9 |
|  | Security man | 4 | 0.7 |
|  | Faculty member | 2 | 0.4 |

**Table 2 (supplementary): Distribution by quartiles of SARS-CoV-2 anti-S titres among 416 unvaccinated SARS-CoV-2 anti-S positive HCWs**

| **Occupation** | **Anti-COVID19 S titre (RU/ml) percentiles** | | | **p-value** |
| --- | --- | --- | --- | --- |
|  | **25^th^** | **50^th^** | **75^th^** |  |
| Physician | 24.0 | 58.0 | >120.0 | 0.254 |
| Nurse | 20.6 | 39.0 | 73.3 |  |
| Technician | 22.0 | 53.3 | 87.7 |  |
| Pharmacist | 15.2 | 25.7 | 67.3 |  |
| Worker | 21.3 | 54.3 | >120.0 |  |
| Office staff | 17.6 | 38.2 | 96.0 |  |
| **Work location** |  |  |  | 0.069 |
| Intensive care units | 28.2 | 47.8 | 110.2 |  |
| Emergency department | 35.9 | 67.2 | >120.0 |  |
| Outpatient clinics | 24.0 | 58.0 | >120.0 |  |
| Wards | 16.1 | 37.3 | 93.2 |  |
| Laboratory | 19.4 | 55.8 | 92.2 |  |
| Radiology | 23.7 | 79.7 | 96.2 |  |
| Intervention room | 12.1 | 32.5 | 89.6 |  |
| Administrative office | 19.6 | 44.3 | 88.4 |  |
| Surgical theatre | 23.2 | 52.7 | >120.0 |  |
| Infection control unit | 21.7 | 112.5 | >120.0 |  |
| Blood bank | 40.7 | 40.7 | 40.7 |  |
| Pharmacy | 15.2 | 25.7 | 49.0 |  |

P: Kruskal-Wallis test
